# Immune characteristics of dedifferentiated retroperitoneal liposarcomas and the reliability of regional samples in evaluating their tumor immune microenvironments

**DOI:** 10.1186/s12957-023-03295-0

**Published:** 2024-01-23

**Authors:** Changsheng Zhou, Ming Li, Yantao Ren, Fenglin Miao, Yue Wang, Ting Wu, Xin Gou, Wengang Li

**Affiliations:** 1 School of Medicine, Xiamen University, Xiamen, Fujian 361102 People’s Republic of China; 2 Department of Hepatobiliary Surgery, Guizhou Provincial People’s Hospital, Guiyang, Guizhou 550002 People’s Republic of China; 3 Department of Hepatobiliary Surgery, Xiang’an Hospital of Xiamen University, School of Medicine, Xiamen University, Xiamen, Fujian 361102 People’s Republic of China; 4 Cancer Research Center of Xiamen University, School of Medicine, Xiamen University, Xiamen, Fujian 361102 People’s Republic of China; 5 Retroperitoneal Tumor Research Center of Oncology Chapter of Chinese Medical Association, School of Medicine, Xiamen University, Xiamen, Fujian 361102 People’s Republic of China; 6 Xiamen Medicine Research Institute, Xiamen, Fujian 361005 People’s Republic of China

**Keywords:** Dedifferentiated retroperitoneal liposarcoma, Immune characteristics, Tumor immune microenvironment, Similarity, Metabolic and PI3K-Akt signaling pathways

## Abstract

**Background:**

Tumor immunotherapy is a new treatment breakthrough for retroperitoneal liposarcoma (RPLS), which is highly invasive and has few effective treatment options other than tumor resection. However, the heterogeneity of the tumor immune microenvironment (TIME) leads to missed clinical diagnosis and inappropriate treatment. Therefore, it is crucial to evaluate whether the TIME of a certain part of the tumor reliably represents the whole tumor, particularly for very large tumors, such as RPLS.

**Methods:**

We conducted a prospective study to evaluate the TIME in different regions of dedifferentiated RPLS (DDRPLS) by detecting the expressions of markers such as CD4^+^, CD8^+^, Foxp3^+^, CD20^+^, CD68^+^, LAMP3^+^, PD-1^+^ tumor-infiltrating lymphocytes (TILs), and PD-L1 in tumors and corresponding paratumor tissues via immunohistochemistry and RNA sequencing.

**Results:**

In DDRPLS, very few TILs were observed. Differentially expressed genes were significantly enriched in cell part and cell functions, as well as the metabolic pathway and PI3K-Akt signaling pathway. In addition, for most tumors (70–80%), the TIME was similar in different tumor regions.

**Conclusions:**

For most tumors (70–80%), the TIME in any region of the tumor reliably represents the whole tumor. DDRPLS may regulate cell functions by modulating the metabolic and PI3K-Akt signaling pathways to promote its malignant behavior.

**Supplementary Information:**

The online version contains supplementary material available at 10.1186/s12957-023-03295-0.

## Introduction

Retroperitoneal liposarcoma (RPLS) is the most common type of retroperitoneal tumor and is characterized by rapid growth and high invasiveness [1]. At present, there is no effective treatment of RPLS except for surgical resection of the tumor [2, 3]. Moreover, surgery often leads to trauma due to the need for extensive resection of several surrounding organs and tissues [4]. In addition, R0 resection is difficult because the tumor is extremely large and has an unclear boundary. Thus, RPLS, especially dedifferentiated RPLS (DDRPLS), has a high recurrence rate and a poor prognosis [5]. Therefore, it is essential to identify new treatment methods.

Tumor immunotherapy has demonstrated positive therapeutic effects in several tumors [6–10]. Therefore, tumor immunotherapy may represent a promising therapeutic approach for RPLS. However, PD-L1 expression varies across tumor regions [11–13], leading to erroneous classification of some PD-L1–positive tumors as PD-L1–negative tumors. In such situations, patients do not receive PD-1/PD-L1 blockers, resulting in the loss of opportunity for possible treatment. Furthermore, the characteristics of the whole tumor cannot be detected. Therefore, evaluation of the tumor immune microenvironment (TIME) before tumor immunotherapy should be based on clinical tumor specimens that accurately reflect the overall tumor characteristics. As a result, it is determined which tumor region accurately reflects the TIME characteristics of the whole tumor. Thus, in this prospective study, we analyzed the TIME characteristics of various tumor regions.

We found that there were very few tumor-infiltrating lymphocytes (TILs) in DDRPLS. Differentially expressed genes (DEGs) were significantly enriched in cell part and cell functions, as well as the metabolic and PI3K-Akt signaling pathways. For most tumors (70–80%), the TIME was similar across different tumor regions. In other words, for most tumors (70–80%), the TIME in any tumor region could reliably represent the whole tumor. DDRPLS may regulate cell functions by modulating the metabolic and PI3K-Akt signaling pathways to promote its malignant behavior.

## Materials and methods

### Specimen collection and clinicopathological data of patients with DDRPLS

**Fig. 1:**
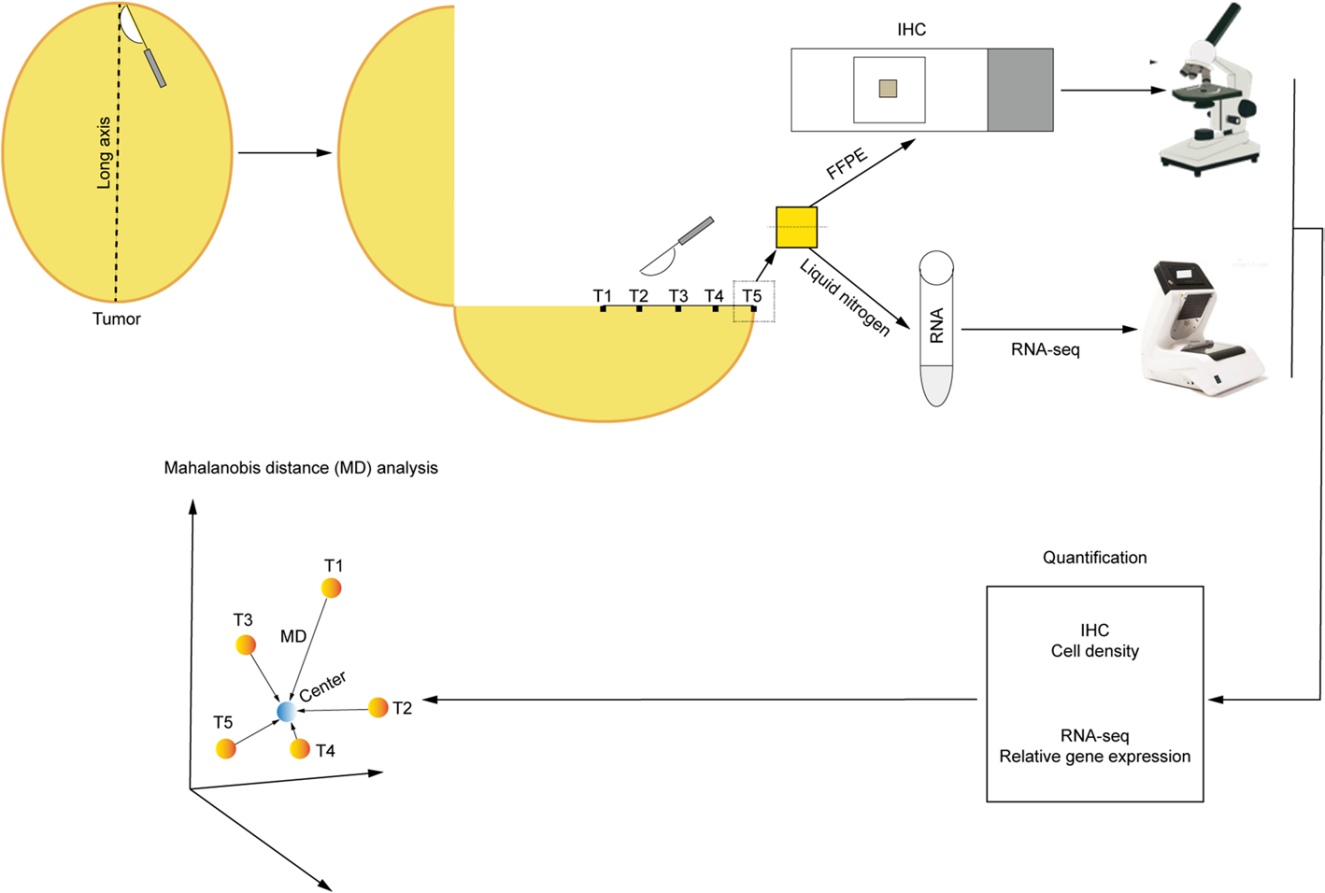
Experimental flowchart. First, the newly resected tumors were cut along their long axis. Next, specimens were obtained from T1-5 sites on the long axis (T1 is the midpoint of the long axis of a tumor; T5 is the outer edge of the long axis of the tumor; T3 is the midpoint of the line between T1 and T5; T2 is the midpoint of the line between T1 and T3; T4 is the midpoint of the line between T3 and T5), and were divided into two groups. In one group, the specimens were placed in 10% formalin for subsequent immunohistochemistry, and in another group, the specimens were placed in liquid nitrogen for subsequent RNA-seq. Finally, the IHC and RNA-seq results were quantified, and the similarity of the TIME in different tumor regions was analyzed using the Mahalanobis distance. FFPE, formalin-fixed and paraffin-embedded; IHC, Immunohistochemistry; and RNA-seq, RNA sequencing

The surgical specimens were obtained from patients with DDRPLS who were treated at Xiang'an Hospital of Xiamen University and the International Hospital of Peking University. All patients provided their written informed consent, and the study protocol was approved by the Ethics Committee of Xiang’an Hospital of Xiamen University. The study was conducted in line with the Declaration of Helsinki. DDRPLS was diagnosed on the basis of standard clinical and histological criteria. All patients underwent tumor resection. Finally, 50 tumor specimens from 10 newly resected tumors and 10 corresponding paratumor tissues were obtained from 10 patients (Fig. 1). The median tumor diameter was 22.75 cm (range, 10–29.9 cm). The clinicopathological data of patients and their tumors are presented in Table 1. Fifty specimens (T1-5) were used for immunohistochemistry (IHC) analysis and 30 specimens (T1, T3, and T5) with corresponding paratumor specimens were used for RNA sequencing (RNA-seq) (Fig. 1).

**Table 1:** Clinicopathological features of patients and their tumors

Case no	Age (years)	Gender	Maximum diameter (cm)	Pathological type	Primary or recurrent	Single or multiple
1	60	M	29.9	CD	P	M
2	66	M	10	CD	R	M
3	52	F	10.9	CD	R	S
4	57	F	25.7	CD	P	S
5	57	F	25.2	PD	R	M
6	63	M	14.8	PD	R	M
7	56	F	11.9	CD	R	S
8	62	M	24.3	PD	P	M
9	57	M	24.3	CD	R	M
10	65	M	21.2	CD	R	M

*M* male, *F* female, *CD* complete dedifferentiation, *PD* partial dedifferentiation,* P* primary tumor, *R* recurrent tumor, *S* single tumor, *M* multiple tumor

RPLS is generally large, which makes it challenging to obtain several samples. Therefore, we hypothesized that RPLS is a round or oval tumor growing from the center to the periphery. In other words, we considered the center and periphery of RPLS to be old and new tissues, respectively. In this case, the largest difference in the TIME within the tumor tissue should be between the center and periphery; that is, if the TIME is similar between the tumor center and periphery, we can infer that the TIME of the whole RPLS is homogeneous. Therefore, we obtained samples from the abovementioned five locations.

### IHC

The tissues were routinely dehydrated, embedded, and sliced. After baking the slices, the sections were successively dewaxed, hydrated, permeated, sealed, antigen-repaired, incubated with primary and secondary antibodies, color-rendered, counterstained, and sealed.

Proportion of positive cells: We randomly obtained 10 high-power vision fields (× 400) from each slice, calculated the number of positive cells among 100 cells in each high-power vision field, and determined the average value from 10 high-power vision fields (positive index, expressed as a percentage). The cells were scored as follows: < 1%, score 0; 1–10%, score 1; 10–50%, score 2; and > 50%, score 3.

Cell density (positive cells/mm^2^): Ten high-power vision fields (× 400; area, 0.0788 mm^2^) were randomly obtained from each slice. We calculated the number of positive cells in each high-power vision field and determined the average value from 10 high-power fields. Supplementary Table S1 presents the information related to antibodies.

### RNA-seq

The total RNA of the samples was extracted using a TRIzol kit (Promega, USA) in line with the manufacturer’s instructions. Residual DNA was removed by digestion with DNase I (TaKaRa Bio, Japan). A260/A280 value was measured to estimate the RNA concentration. A small quantity of RNA product was for agarose gel electrophoresis to determine the RNA quality. Next, the qualified RNAs were quantified using Qubit3.0 with the Qubit™ RNA Broad Range Assay kit (Life Technologies, Q10210). A total of 2 μg total RNA was used for stranded RNA sequencing library preparation using KC™ Stranded mRNA Library Prep Kit for Illumina® (Wuhan Seqhealth, China) in accordance with the manufacturer’s instructions. PCR products with 200–500 bps were enriched, quantified, and finally sequenced on a Novaseq 6000 sequencer (Illumina) using a PE150 model. Raw sequencing data were filtered using Trimmomatic (version 0.36). Low-quality reads were discarded, and the reads contaminated with adaptor sequences were trimmed. Reads mapped to the exon regions of each gene were counted using featureCounts (Subread-1.5.1; Bioconductor). The abundance of each immune cell type was estimated as the average expression level of the corresponding genes [14] (Supplementary Table S2).

### Statistical analysis

Differences between groups, correlations between factors, and the similarity of the TIME in different tumor regions were analyzed using the Mann–Whitney *U* test, Spearman’s correlation coefficients, and Mahalanobis distance analysis on SPSS version 23.0 (SPSS Inc., IL, USA) and GraphPad Prism software version 8.0 (GraphPad Software Inc., San Diego, CA, USA). Statistical significance was defined as *p* < 0.05.

Mahalanobis distance is the covariance distance in data. It is used to effectively determine the similarity between two unknown sample sets and to effectively evaluate the outliers and similarities among individuals in a population [14]. Additionally, compared with the common Euclidean distance, Mahalanobis distance has two advantages. First, its scale remains unchanged, and thus the differences in unit measurement values of different biomarkers will not affect the analysis results. Second, it also evaluates correlations between covariates, which can capture not only the difference in a single variable but also the differences between a group of variables[15]. In summary, Mahalanobis distance analysis is an ideal method for evaluating the similarity between unknown sample sets [14].

## Results

### Characteristics of TIME in DDRPLS

**Fig. 2:**
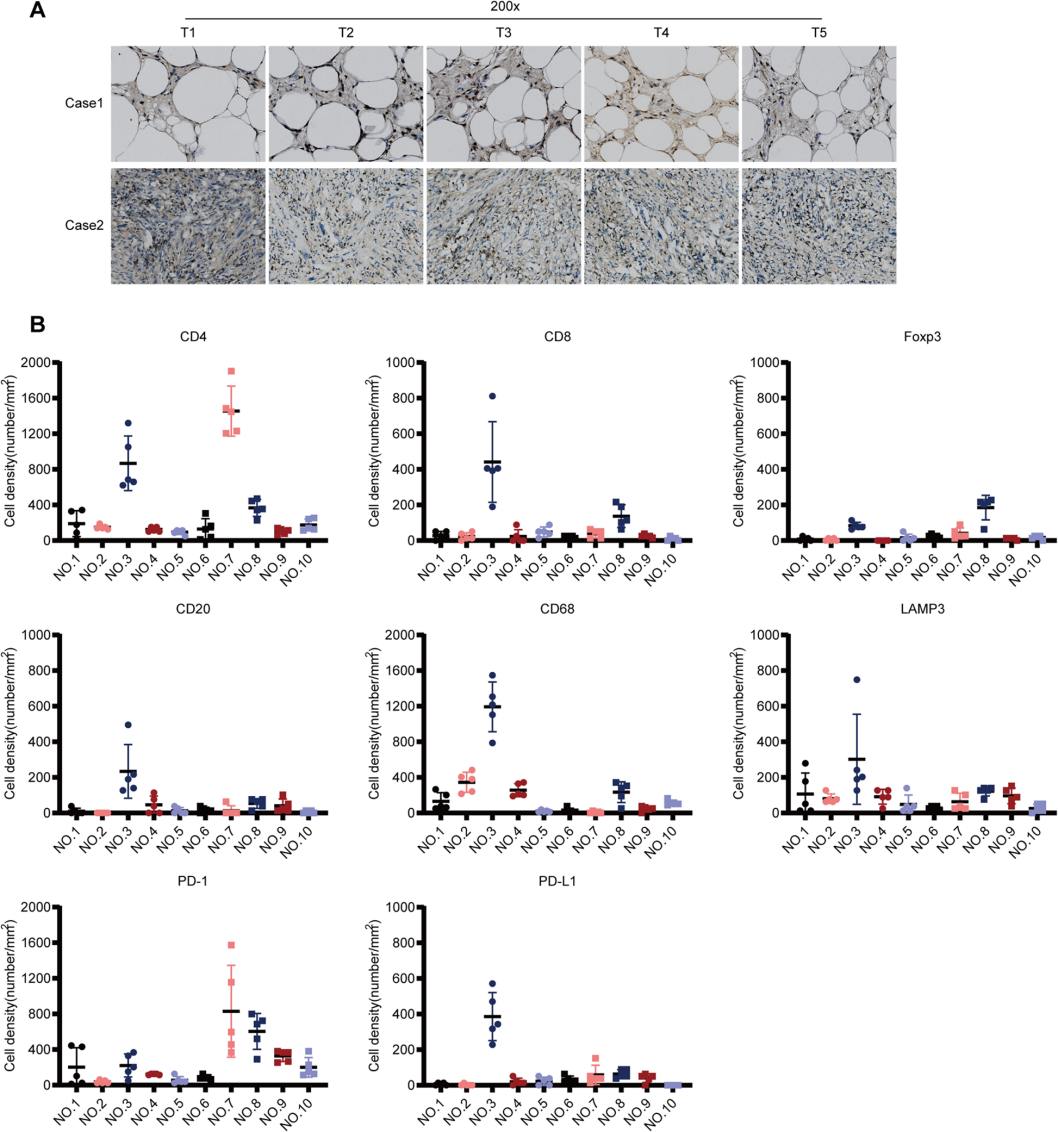
TIME of DDRPLS.** A** The TIME between different patients was heterogeneous; however, TIME in different tumor regions was similar (IHC, represented by CD4). **B** Immune cell constitution in DDRPLS using IHC data. For most tumors, the distribution range of immune cells was narrow for different tumor regions. Each dot denotes the immune cell density of a sample. TIME, tumor immune microenvironment; DDRPLS, dedifferentiated retroperitoneal liposarcoma; and IHC, immunohistochemistry

To analyze the characteristics of the TIME in different tumor regions, we first detected the expression of immune markers including CD4^+^, CD8^+^, Foxp3^+^, CD20^+^, CD68^+^, LAMP3^+^, PD-1^+^ TILs, and PD-L1 in different tumor regions using IHC. As shown in Fig. 2A, the TIME was significantly heterogeneous between patients. However, the TIME was similar in different tumor regions (Fig. 2A). Furthermore, for most tumors, the distribution range of immune cells in different tumor regions was narrow (Fig. 2B, Supplementary Fig. 1), indicating similar TIME in these regions.

**Fig. 3:**
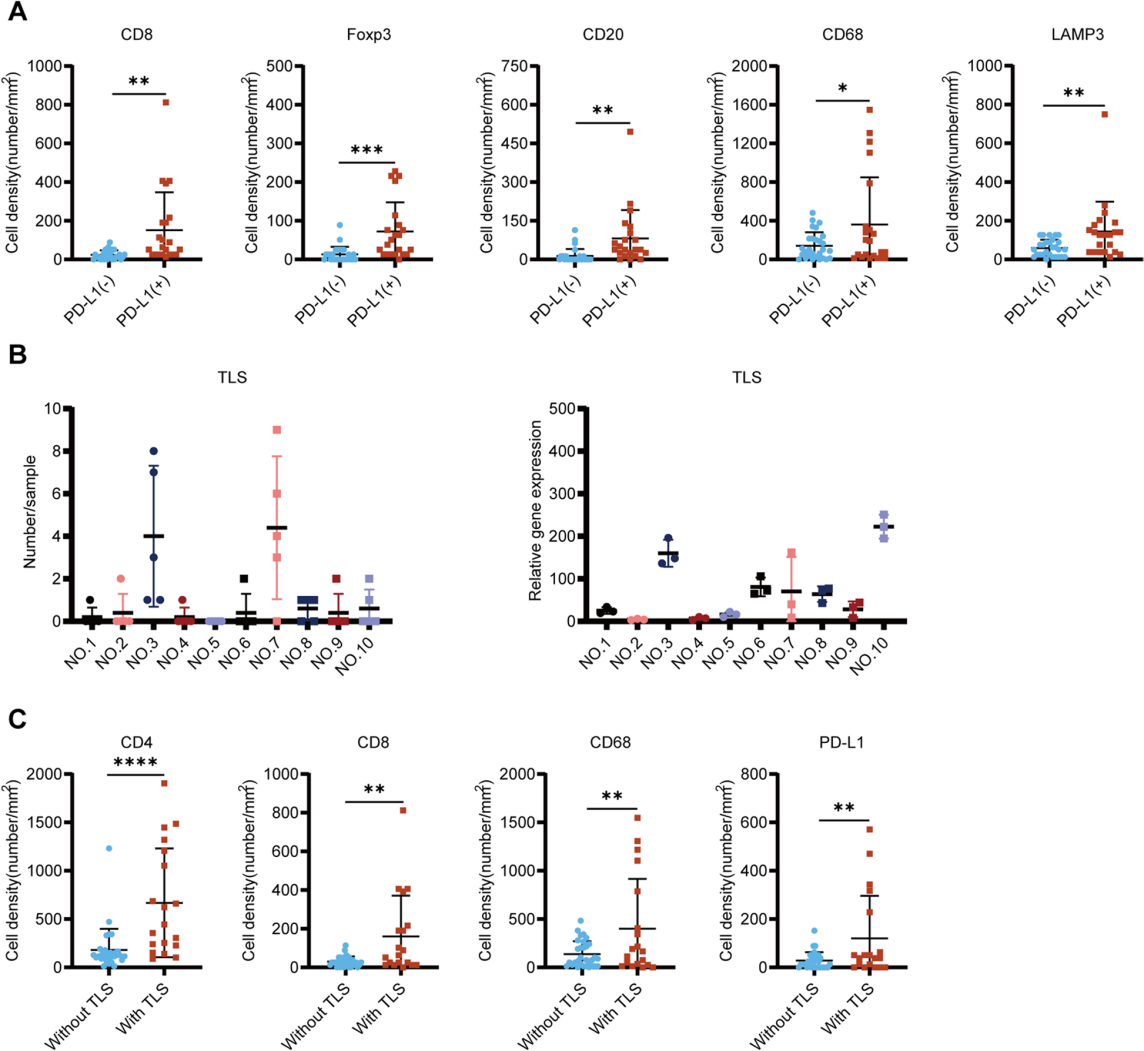
PD-L1 and TLS in DDRPLS. **A** PD-L1 expression increases CD8^+^ T cells, Foxp3^+^ Tregs, CD20^+^ B cells, CD68^+^ macrophages, and LAMP3^+^ DCs. **B** Number or relative gene expression of TLS per sample (left: IHC data; right: RNA-seq data). Each dot denotes the number or relative gene expression of a sample. **C** TLS increases CD4^+^ T cells, CD8^+^ T cells, CD68^+^ macrophages, and PD-L1^+^ cells in DDRPLS. *p < 0.05, **p < 0.01, ***p < 0.001, ****p < 0.0001. TLS, tertiary lymphatic structure; DDRPLS, dedifferentiated retroperitoneal liposarcoma; DCs, dendritic cells; IHC, immunohistochemistry; and RNA-seq, RNA sequencing

In addition, very few TILs were observed in DDRPLS (Fig. 2, Supplementary Figures S1–S4), which is consistent with a previous report [16]. CD4^+^ T cells were the most common, followed by CD68^+^ macrophages and PD-1^+^ cells, while Foxp3^+^ Tregs were the least common, followed by CD8^+^ T cells and PD-L1^+^ cells (Supplementary Figures S2–S4). Interestingly, PD-1^+^ cells were found in all DDRPLS specimens (Table 2). However, PD-L1^+^ cells were very rare and not found in many specimens (Table 3). Additionally, PD-L1 expression was closely related to other TILs and was associated with increased numbers of CD8^+^ T cells, Foxp3^+^ Tregs, CD20^+^ B cells, CD68^+^ macrophages, and LAMP3^+^ dendritic cells (DCs) (Fig. 3A).

**Table 2:** PD-1 expression

Tumor no	Number of samples	Score 0	Score 1	Score 2	Score 3
		Number (%)	Number (%)	Number (%)	Number (%)
1	5			1(20%)	4(80%)
2	5			2(40%)	3(60%)
3	5				5(100%)
4	5				5(100%)
5	5				5(100%)
6	5				5(100%)
7	5			2(40%)	3(60%)
8	5				5(100%)
9	5				5(100%)
10	5			4(80%)	1(20%)

**Table 3:** PD-L1 expression

Tumor no	Number of samples	Score 0	Score 1	Score 2	Score 3
		Number (%)	Number (%)	Number (%)	Number (%)
1	5	3(60%)	1(20%)	1(20%)	
2	5	5(100%)			
3	5				5(100%)
4	5	5(100%)			
5	5	2(40%)	1(20%)	2(40%)	
6	5	1(20%)	1(20%)	1(20%)	2(40%)
7	5	5(100%)			
8	5		3(60%)	2(40%)	
9	5	2(40%)	2(40%)	1(20%)	
10	5	5(100%)

The tertiary lymphoid structure (TLS) is the region of lymphocyte aggregation [17, 18], and it can provide an important functional environment for cellular and humoral immunity to maintain and promote the antitumor immune response [17–19]. A higher number of TLS is associated with a stronger antitumor immune response [20, 21]. Therefore, to analyze TLS in the TIME of DDRPLS, the number of TLS per tissue section was determined. As shown in Fig. 3B, 56 TLSs were found in 50 regions of 10 tumors. The highest number of TLS found in one region was 9. Interestingly, compared with tumors without TLS, the numbers of CD4^+^ T cells, CD8^+^ T cells, CD68^+^ macrophages, and PD-L1^+^ cells in the tumors with TLS were significantly increased (Fig. 3C).

### Relationships between immune characteristics and clinicopathological features of DDRPLS patients

As shown in Supplementary Figures S5 and S6 and Supplementary Table S3, the immune characteristics of tumors were related to the clinicopathological features of DDRPLS patients. Higher numbers of CD4^+^ T cells, CD8^+^ T cells, CD20^+^ B cells, CD68^+^ macrophages, LAMP3^+^ DCs, PD-L1^+^ cells, and TLSs were detected in patients with a single tumor than in those with multiple tumors (Supplementary Figure S5). The density of CD4^+^ T cells (*r* =  − 0.5187, *p* = 0.0001), CD8^+^ T cells (*r* =  − 0.3221, *p* = 0.0225), CD68^+^ macrophages (*r* =  − 0.4097, *p* = 0.0031), and PD-L1^+^ cells (*r* =  − 0.4119, *p* = 0.003), and the number of TLSs (*r* =  − 0.4665, *p* = 0.0006), were negatively correlated with tumor size (Supplementary Figure S6). However, no significant correlation was found between immune characteristics and diagnostic status (primary or recurrent tumor) (Supplementary Table S3). In addition, higher densities of CD8^+^ T cells (*p* = 0.0193), Foxp3^+^ Tregs (*p* = 0.0029), CD68^+^ macrophages (*p* = 0.0028), and PD-L1^+^ cells (*p* = 0.0439) were detected in older patients than in younger patients. Furthermore, higher densities of CD4^+^ T cells (*p* = 0.0003), CD8^+^ T cells (*p* = 0.0188), CD20^+^ B cells (*p* = 0.0193), CD68^+^ macrophages (*p* = 0.03), and PD-L1^+^ cells (*p* = 0.0034), and a greater number of TLSs (*p* = 0.0044), were detected in female patients than in male patients (Supplementary Table S3).

### Similarities in TIME between different regions of DDRPLS

**Fig. 4:**
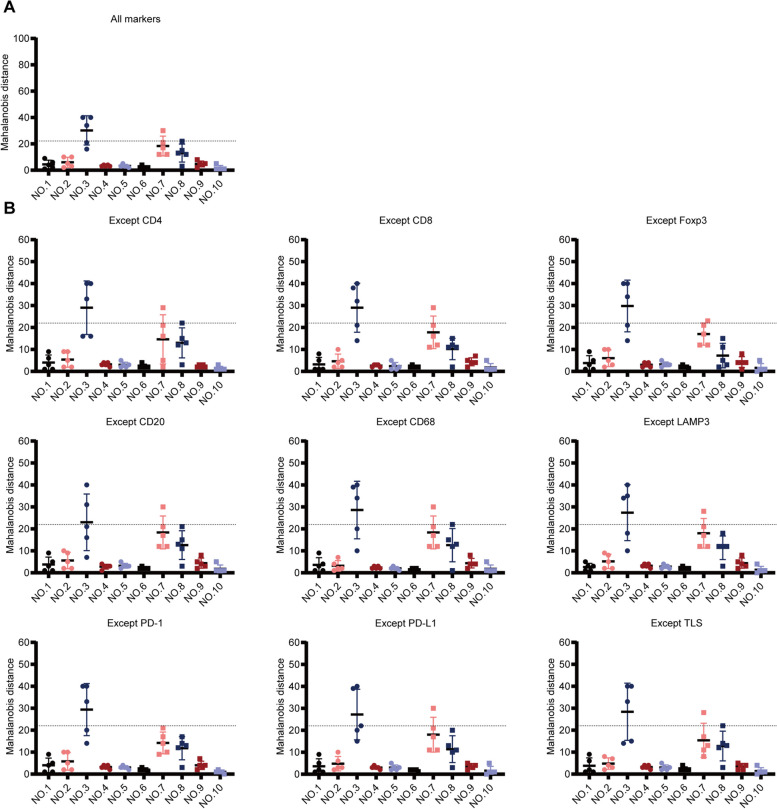
Similarity of the TIME across different tumor regions using IHC data. **A** Mahalanobis distance of all immune markers (including eight immune cells and TLS). **B** Mahalanobis distance of all immune markers except one. Each dot denotes the Mahalanobis distance of a region. Dotted lines denote the critical value of Mahalanobis distance. TIME, tumor immune microenvironment; IHC, immunohistochemistry; and TLS, tertiary lymphatic structure

The abovementioned results demonstrated that the TIME was similar in different regions of DDRPLS. To further confirm this finding, IHC data were analyzed to determine the Mahalanobis distance. As shown in Figure 4A, for most tumors, the TIME was similar across different tumor regions. In 7 of 10 tumors (70%; nos. 1, 2, 4, 5, 6, 9, and 10), the distribution range of Mahalanobis distance was narrow for different tumor regions, while in 3 of 10 tumors (30%; nos. 3, 7, and 8), the distribution range of Mahalanobis distance was wide for different tumor regions (Fig. 4A). In addition, in 8 of 10 tumors (80%; nos. 1, 2, 4, 5, 6, 8, 9, and 10), the Mahalanobis distance for all tumor regions was 0–22 (critical value), while it was more than 22 (critical value) in 2 of 10 tumors (20%; Nos. 3 and 7) (Fig. 4A). These data indicated that for most tumors (70–80%), the TIME was similar for different tumor regions.

To analyze the factors affecting the similarity of TIME across different tumor regions, Mahalanobis distance was calculated again after removing markers one by one. As shown in Fig. 4B, after removing CD8^+^ T cells, Foxp3^+^ Tregs, LAMP3^+^ DCs, and PD-1^+^ cells, the Mahalanobis distance for different tumor regions (no. 8) was significantly narrowed, indicating that CD8^+^ T cells, Foxp3^+^ Tregs, LAMP3^+^ DCs, and PD-1^+^ cells were the main influencing factors leading to the TIME heterogeneity in different tumor regions (no. 8). For tumor no. 3, not only the distribution range of the Mahalanobis distance for different tumor regions was wide, but also the Mahalanobis distance did not decrease to less than 22 (critical value), regardless of removing the markers (Fig. 4B). This indicated that the TIME was highly heterogeneous across the different regions of tumor no. 3. However, for tumor No. 7, the Mahalanobis distance in all regions decreased to less than 22 (critical value) after removing PD-1^+^ cells (Fig. 4B), indicating that PD-1^+^ cells were the main influencing factor leading to the heterogeneity in the TIME across different regions of tumor no. 7.

**Fig. 5:**
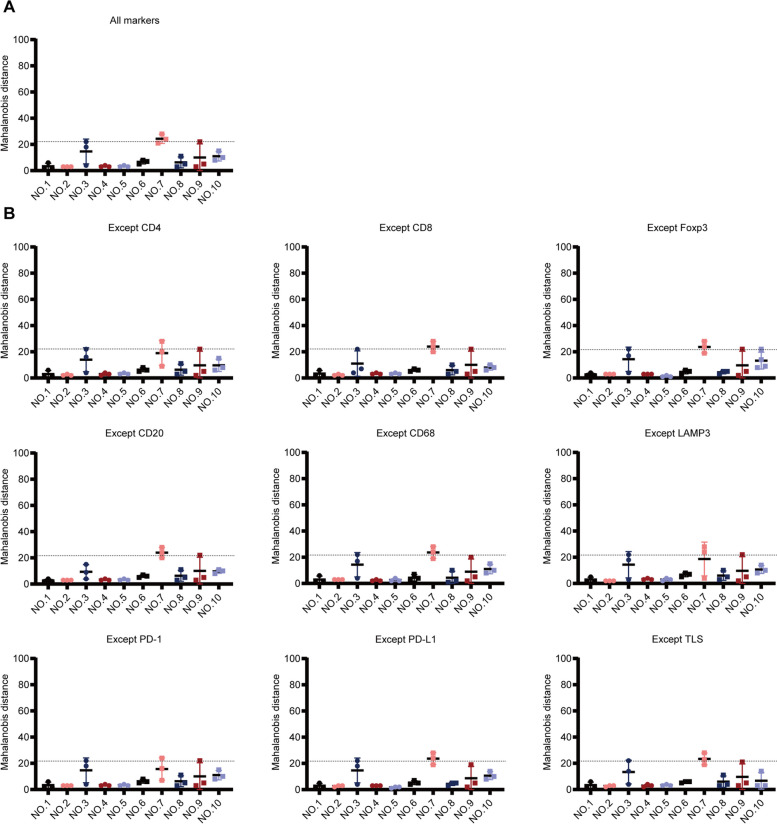
Similarity of the TIME across different tumor regions using RNA-seq data.** A** Mahalanobis distance of all immune markers (including eight immune cells and TLS). **B** Mahalanobis distance of all immune markers except one. Each dot denotes the Mahalanobis distance of a region. Dotted lines denote the critical value of Mahalanobis distance. TIME, tumor immune microenvironment; RNA-seq, RNA sequencing; and TLS, tertiary lymphatic structure

RNA-seq results showed that in 8 of 10 tumors (80%; nos. 1, 2, 4, 5, 6, 7, 8, and 10), the distribution range of the Mahalanobis distance was narrow for different tumor regions, while in 2 of 10 tumors (20%; nos. 3 and 9), the distribution range of the Mahalanobis distance was wide for different tumor regions (Fig. 5A). Furthermore, in 8 of 10 tumors (80%; nos. 1, 2, 4, 5, 6, 8, 9, and 10), the Mahalanobis distance for all tumor regions was 0–22 (critical value), and in 2 of 10 tumors (20%; nos. 3 and 7), the Mahalanobis distance for some tumor regions exceeded 22 (critical value) (Fig. 5A). These data indicated that for most tumors (80%), the TIME was similar for different tumor regions. Similarly, for tumor no. 3, the distribution range of the Mahalanobis distance in different regions was narrowed, whereas the Mahalanobis distance in all regions was decreased to less than 22 (critical value) after removing CD20^+^ B cells (Fig. 5B), indicating that CD20^+^ B cells were the main influencing factor leading to the heterogeneity in TIME among different regions of the tumor no. 3. However, for tumor No. 7, the Mahalanobis distance for some regions did not decrease to less than 22 (critical value), regardless of removing the markers (Fig. 5B), indicating that the TIME was highly heterogeneous for different regions of the tumor no. 7. In addition, for tumor no. 9, regardless of removing any of the markers, the distribution range of the Mahalanobis distance was not narrowed for different regions, although they were all within 22 (critical value) (Fig. 5B).

As shown in Supplementary Figure S7, there was a strong correlation between IHC and RNA-seq data for the evaluation of TIME. For CD4^+^ T cells, CD8^+^ T cells, CD20^+^ B cells, LAMP3^+^ DCs, and PD-1^+^ cells, RNA-seq was positively correlated with IHC (Supplementary Figure S7). In addition, the IHC results showed that in 7 of 10 tumors (70%; nos. 1, 2, 4, 5, 6, 9, and 10), the distribution range of the Mahalanobis distance was narrow for different tumor regions, and in 3 of 10 tumors (30%; nos. 3, 7, and 8), the distribution range of the Mahalanobis distance was wide for different tumor regions (Supplementary Figure S8A). RNA-seq results showed that in 8 of 10 tumors (80%; nos. 1, 2, 4, 5, 6, 7, 8, and 10), the distribution range of the Mahalanobis distance was narrow for different tumor regions, and in 2 of 10 tumors (20%; Nos. 3 and 9), the distribution range of the Mahalanobis distance was wide for different tumor regions(Supplementary Figure S8B). Moreover, both IHC and RNA-seq results showed that in 8 of 10 tumors (80%; nos. 1, 2, 4, 5, 6, 8, 9, and 10), the Mahalanobis distance in all tumor regions was within 22 (critical value), and in 2 of 10 tumors (20%; Nos. 3 and 7), the Mahalanobis distance for some tumor regions was more than 22 (critical value) (Supplementary Figure S8A, B). These data indicated good correlation and consistency between IHC and RNA-seq data for the evaluation of TIME in DDRPLS.

Interestingly, for regions used for RNA-seq detection (T1, T3, and T5), IHC results showed that in 9 of 10 tumors (90%; nos. 1, 2, 3, 4, 5, 6, 7, 9, and 10), the distribution range of the Mahalanobis distance was narrow for different tumor regions, and in 1 of 10 tumors (10%; no. 8), the distribution range of the Mahalanobis distance was wide for different tumor regions(Supplementary Figure S8C). RNA-seq results showed that in 8 of 10 tumors (80%; nos. 1, 2, 4, 5, 6, 7, 8, and 10), the distribution range of the Mahalanobis distance was narrow for different tumor regions, and in 2 of 10 tumors (20%; Nos. 3 and 9), the distribution range of the Mahalanobis distance was wide for different tumor regions (Supplementary Figure S8B). Moreover, both IHC and RNA-seq results showed that in 8 of 10 tumors (80%; nos. 1, 2, 4, 5, 6, 8, 9, and 10), the Mahalanobis distance in all tumor regions was within 22 (critical value), and in 2 of 10 tumors (20%; Nos. 3 and 7), the Mahalanobis distance in some tumor regions was more than 22 (critical value) (Supplementary Figure S8B, C). These data also indicated very good consistency between IHC and RNA-seq data for evaluating the TIME of DDRPLS.

### Characteristics of related pathways in DDRPLS

**Fig. 6:**
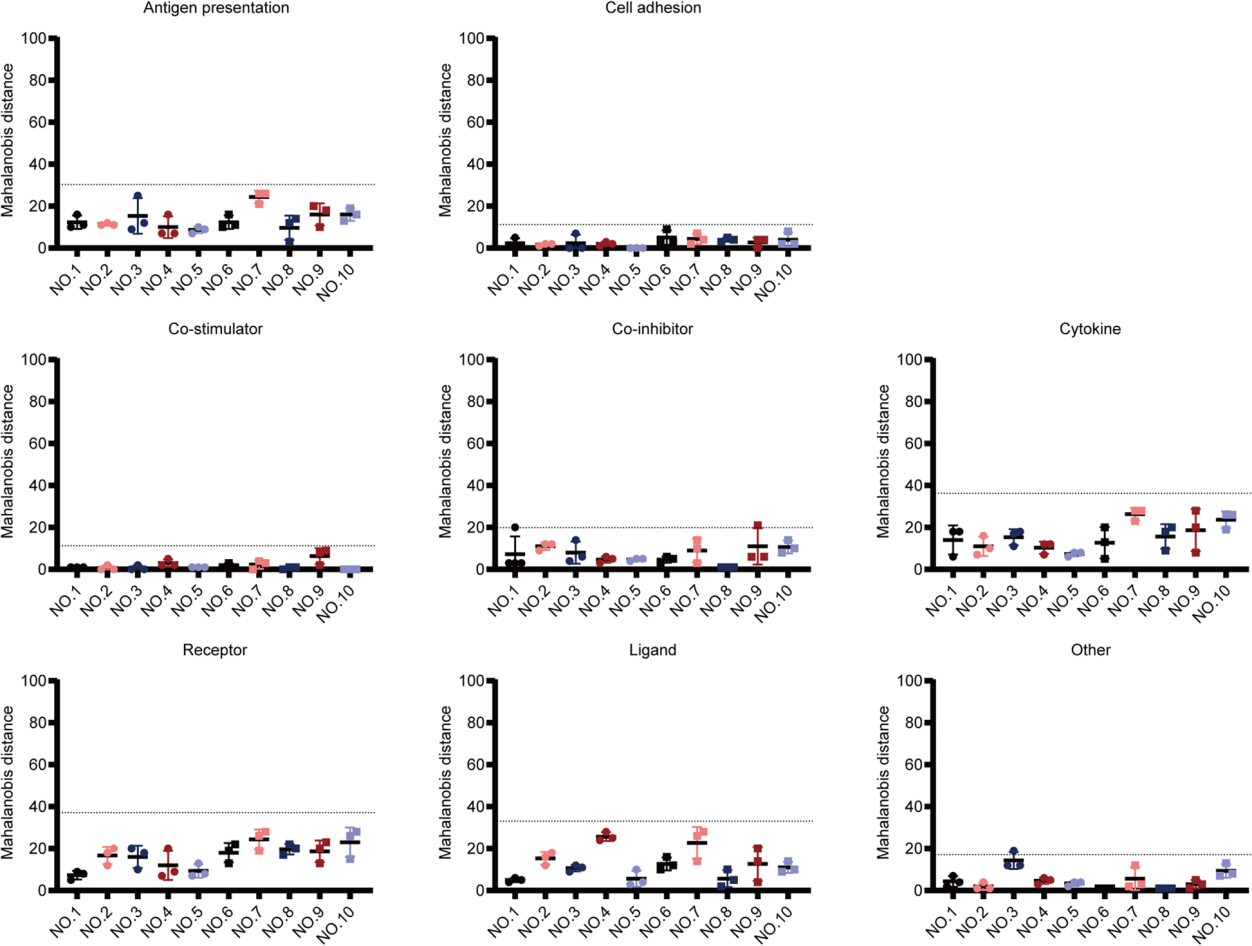
Similarity of the immune-related pathways in different tumor regions using RNA-seq data alone. Immune-related pathways in different tumor regions were similar. Each dot denotes the Mahalanobis distance of a region. Dotted lines denote the critical value of Mahalanobis distance. RNA-seq, RNA sequencing

The abovementioned results showed very good correlation and consistency between IHC and RNA-seq data for evaluating the TIME of DDRLPS. Moreover, RNA-seq data were more comprehensive and extensive than IHC data. Thus, we used RNA-seq data alone to analyze immune-related pathways in the TIME of DDRPLS, including antigen presentation, cell adhesion, co-stimulator, co-inhibitor, cytokine, receptor, and ligand [22] (Fig. 6, Supplementary Figures S9 and S10). The results showed that immune-related pathways in different tumor regions were also similar (Fig. 6, Supplementary Figure S10).

Gene Ontology (GO) enrichment analysis of DEGs in the tumor and corresponding paratumor tissues detected via RNA-seq showed that, compared to paratumor tissues, DEGs in DDRPLS were significantly enriched in the intracellular part, intracellular, cellular process, cell part, cell, binding, and single organism process functions, with cell part and cell functions exhibiting especially significant enrichment in almost all DDRPLS (Supplementary Figure S11). These findings suggest that DDRPLS may undergo malignant transformation primarily through the regulation of cell parts and cell functions.

**Fig. 7:**
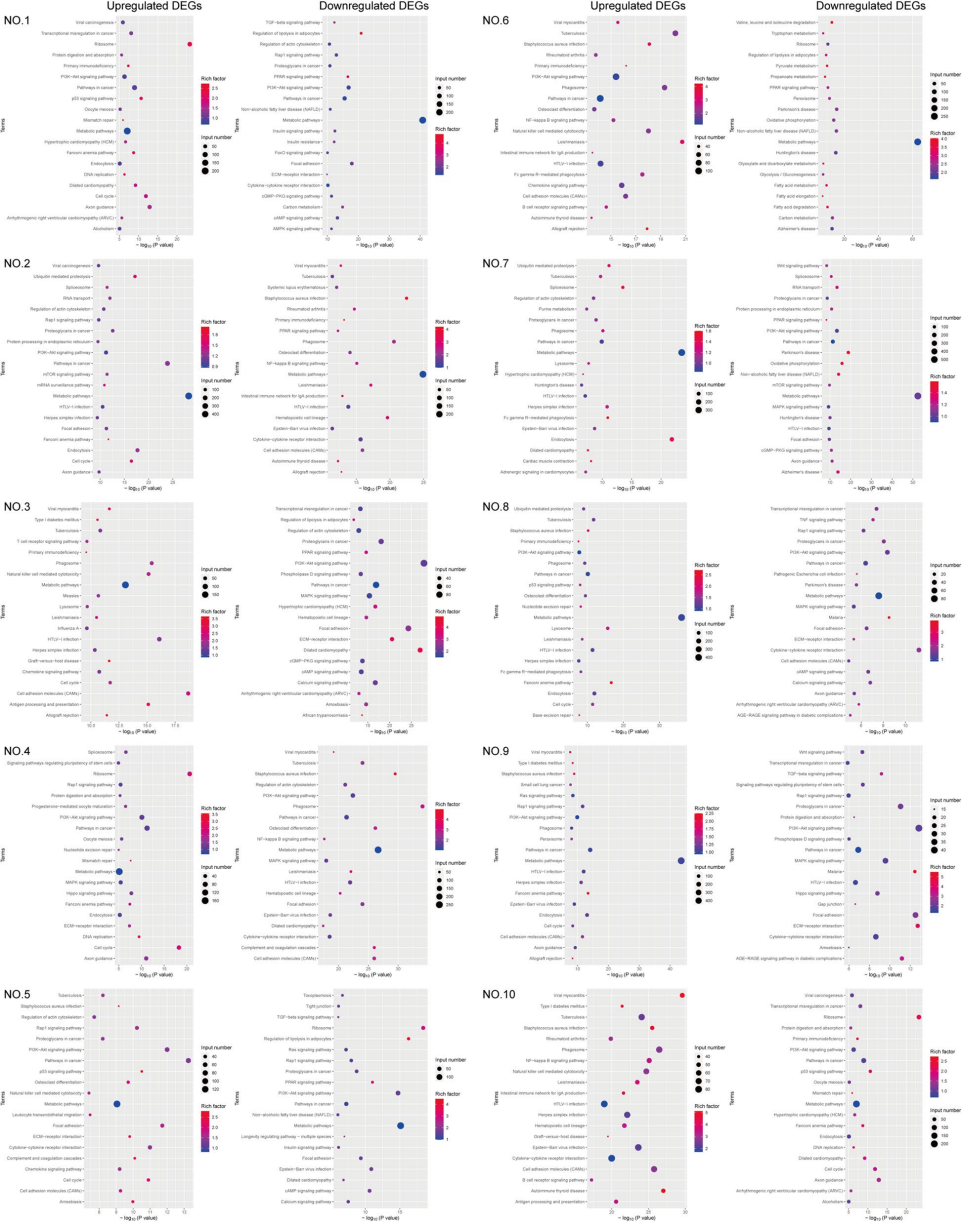
KEGG pathway enrichment analysis of DEGs in DDRPLS. Compared to paratumor tissues, DEGs in DDRPLS were significantly enriched in several pathways, such as the ribosome, metabolic, PI3K-Akt signaling, cell adhesion molecules (CAMs), phagosome, and cancer pathways, with the most significant enrichment in the metabolic pathway and the PI3K-Akt signaling pathway. KEGG, Kyoto Encyclopedia of Genes and Genomes; DEGs, differentially expressed genes; DDRPLS, dedifferentiated retroperitoneal liposarcoma

In addition, further Kyoto Encyclopedia of Genes and Genomes (KEGG) pathway enrichment analysis of DEGs in the tumor and corresponding paratumor tissues showed that, compared to paratumor tissues, DEGs in DDRPLS were mainly enriched in pathways such as ribosomes, metabolic pathway, PI3K-Akt signaling pathway, cell adhesion molecules (CAMs), phagosome, and pathways in cancer (Fig. 7). The metabolic pathway and PI3K-Akt signaling pathway exhibited especially significant enrichment in almost all DDRPLS (Fig. 7). These findings suggest that DDRPLS may regulate cell functions primarily by regulating the metabolic and PI3K-Akt signaling pathways, leading to malignant transformation.

## Discussion

In this study, we showed that in DDRPLS, the number of TILs was very low, and CD4^+^ T cells were the most common, while Foxp3^+^ Tregs were the least common. DEGs were significantly enriched in cell part and cell functions, as well as the metabolic and PI3K-Akt signaling pathways. For most tumors (70–80%), the TIME in different tumor regions was similar.

Studies evaluating the TIME have mainly focused on inter-patient or inter-tumor heterogeneity and the relationship between the TIME and clinicopathological features of patients [23–33]. Few studies have focused on intratumor similarity [34, 35], especially for very large tumors such as RPLS. The PD-L1 expression [11–13] varies across different regions of some tumors, which may lead to misclassification of some PD-L1–positive tumors as PD-L1–negative tumors, hindering possible treatment with PD-1/PD-L1. Therefore, in both clinical and experimental research, we often need to obtain tumor specimens to reliably reflect the TIME of the whole tumor, especially for large tumors, such as RPLS. Therefore, to overcome this challenge and provide guidance for clinical diagnosis and treatment, the characteristics of the TIME in different tumor regions were investigated. The results showed that for most tumors (70–80%), the TIME was similar in different tumor regions. In other words, for most tumors (70–80%), the obtained specimen can reliably represent the TIME of the whole tumor, regardless of the region it is obtained from. Therefore, for most tumors (70–80%), the TIME of any tumor region can reliably represent the whole tumor.

However, we found two tumors (nos. 3 and 7) using IHC and two tumors (nos. 3 and 7) using RNA-seq that displayed significant heterogeneity in different tumor regions, for which the TIME in any tumor region could not reliably represent the whole tumor. We thought that the heterogeneity had little to do with tumor size or type, but was related to the inherent tumor characteristics. These results are supported by a study that demonstrated that different tumor regions might undergo different stages of immune-editing of neoantigen-harboring cancer cells [36].

In addition, our results showed that the TIME of DDRPLS contained very few TILs, which is consistent with a previous report [16]. This might be related to the large volume of tumor cells, as they occupy a large space in the tumor, especially well-differentiated liposarcoma (data not shown), which might explain why many reports have shown that tumor immunotherapy was ineffective for well-differentiated liposarcoma [37, 38]. These findings also indicate that it is essential to develop strategies to increase immune cells for tumor immunotherapy of RPLS. Similarly, TILs in multiple tumors are reduced because many of these tumors are partially dedifferentiated (Table 1) with a large number of tumor cells.

Moreover, we found that the number of TLSs was associated with the number of CD4^+^T cells, CD8^+^ T cells, CD20^+^ B cells, and CD68^+^ macrophages. In other words, in tumors with a greater number of TLSs, the density of CD4^+^T cells, CD8^+^ T cells, CD20^+^ B cells, and CD68^+^ macrophages were also higher. These phenomena were mainly related to strong anti-tumor immune responses [21, 39]. In addition, compared to multiple and large tumors, the number of TLSs was greater and the density of CD4^+^T cells, CD8^+^ T cells, CD20^+^ B cells, and CD68^+^ macrophages was higher in single and small tumors. These findings indicate that, in patients with high levels of TLSs, CD4^+^T cells, CD8^+^ T cells, CD20^+^ B cells, and CD68^+^ macrophages, tumor growth may be inhibited.

Furthermore, to avoid the unreliability of a single method, such as IHC alone, we evaluated the correlation and consistency between IHC and RNA-seq for evaluating the TIME. Interestingly, our results showed a very good correlation and consistency between IHC and RNA-seq for evaluating the TIME. Moreover, compared to the IHC data, RNA-seq data were more comprehensive and extensive. These findings indicate that the use of RNA-seq data alone to evaluate the TIME is feasible and comprehensive.

Our study also had some limitations. First, the sample size was relatively small, although it was consistent with statistical principles and the sample size of many previous studies [40–42]. Therefore, it is necessary to expand the sample size in future studies. Second, conventional IHC cannot fully characterize the functional status of immune cells and fully detect the components of TIME, such as naive, effector, memory, or exhausted T cells, M1 or M2 polarized macrophages, and granulocytic or monocytic myeloid-derived suppressor cells. Ideal evaluation methods applicable to FFPE specimens include multiplexed fluorescent IHC [35] and mass spectrometry-based multiplexed ion beam imaging [43, 44]. However, they are not routinely used in the clinical setting and are very expensive. The correlation and consistency between RNA-seq and IHC for evaluating the TIME were very good, and RNA-seq data were very comprehensive and extensive. Therefore, RNA-seq can be used alone to evaluate the TIME in a future larger study. Third, this study suggested that DDRPLS may regulate cell functions by modulating the metabolic and PI3K-Akt signaling pathways to promote its malignant behavior. However, this needs to be further investigated in the future.

## Conclusions

In this study, very few TILs were found in DDRPLS. DEGs were significantly enriched in cell part and cell functions, as well as the metabolic and PI3K-Akt signaling pathways. For most tumors (70–80%), the TIME in different tumor regions was similar. Therefore, the TIME in any tumor region could reliably represent the whole tumor. DDRPLS may regulate cell functions by modulating the metabolic and PI3K-Akt signaling pathways to promote its malignant behavior. This is important to consider when obtaining a tumor specimen to reliably represent the TIME of the whole tumor in clinical or experimental research. Furthermore, these findings may provide guidance for the clinical diagnosis and treatment, as well as for research into the mechanism of RPLS.

### Supplementary Information

**Additional file 1: Supplementary Table S1. **Antibody information. **Supplementary Table S2.** All immune markers corresponding genes. **Supplementary Table S3.** Correlation between immune markers and clinicopathological features of patients.

**Additional file 2: Supplementary Figure 1. **Immune cell constitution in DDRPLS using RNA-seq data. For most tumors, the distribution range of immune cells was narrow for different tumor regions. Each dot denotes the relative gene expression of a sample. DDRPLS, Dedifferentiated retroperitoneal liposarcoma; RNA-seq, RNA sequencing. **Supplementary Figure 2.** Immune cell constitution in the same region of DDRPLS. Very few immune cells were observed in DDRPLS. A: Immune cells in the same region of DDRPLS (IHC, represented by T3). B: Immune cell density in the same region of DDRPLS (represented by T3). Each dot denotes a tumor. C: Immune cell proportion in the same region of DDRPLS (represented by T3). Each dot denotes a tumor. DDRPLS, Dedifferentiated retroperitoneal liposarcoma; IHC, Immunohistochemistry. **Supplementary Figure 3.** Representative IHC images of immune cells in DDRPLS. Very few immune cells were observed in DDRPLS. IHC, Immunohistochemistry; DDRPLS, Dedifferentiated retroperitoneal liposarcoma. **Supplementary Figure 4.** Immune cell constitution in the same region of DDRPLS. Very few immune cells were observed in DDRPLS. A: Immune cell density in the same region of DDRPLS (represented by T3). B: Immune cell proportion in the same region of DDRPLS (represented by T3). DDRPLS, Dedifferentiated retroperitoneal liposarcoma. **Supplementary Figure 5.** Relationship between immune markers and tumor type in DDRPLS. Fewer immune markers were detected in multiple tumorsa single tumor. *p < 0.05, **p < 0.01, ***p < 0.001, ****p < 0.0001. DDRPLS, Dedifferentiated retroperitoneal liposarcoma; TLS, Tertiary lymphatic structure. **Supplementary Figure 6.** Relationship between immune markers and tumor size in DDRPLS. Immune markers were negatively correlated with tumor size in DDRPLS. DDRPLS, Dedifferentiated retroperitoneal liposarcoma; TLS, Tertiary lymphatic structure. **Supplementary Figure 7.** Correlation between IHC and RNA-seq  data for the evaluation of TIME. RNA-seq was positively correlated with IHC data for the evaluation of TIME. IHC, Immunohistochemistry; RNA-seq, RNA sequencing; and TIME, Tumor immune microenvironment. **Supplementary Figure 8.** Consistency between IHC and RNA-seq for evaluating TIME. IHC and RNA-seq data demonstrated consistent results for the evaluation of TIME. A: Mahalanobis distance of all regions (T1-5) used for IHC. B: Mahalanobis distance of all regions used for RNA-seq. C: Mahalanobis distance of regions (T1, T3, and T5) used for RNA-seq (detected by IHC). Each dot denotes the Mahalanobis distance of a region. Dotted lines denote the critical value of Mahalanobis distance. IHC, Immunohistochemistry; RNA-seq, RNA sequencing; and TIME, Tumor immune microenvironment.** Supplementary Figure 9.** Relative expression of immune-related pathways in DDRPLS. The relative expression of immune-related pathways in DDRPLS was low (represented by T3). DDRPLS, Dedifferentiated retroperitoneal liposarcoma. **Supplementary Figure 10. **Similarity of cytokines in different tumor regions of using RNA-seq data alone. Cytokines in different tumor regions were similar. Each dot denotes the Mahalanobis distance of a region. Dotted lines denote the critical value of Mahalanobis distance. RNA-seq, RNA sequencing. **Supplementary Figure 11.** GO enrichment analysis of DEGs in DDRPLS. Compared withto paratumor tissues, DEGs in DDRPLS were significantly enriched in the intracellular part, intracellular, cellular process, cell part, cell, binding, and single organism process functions, with the most significant enrichment in the cell part and cell functions. GO, Gene Ontology; DEGs, Differentially expressed genes; DDRPLS, Dedifferentiated retroperitoneal liposarcoma.

## Data Availability

The data and materials of this study are available from the corresponding author and/or the first author on reasonable request.

## Abbreviations

RPLS: Retroperitoneal liposarcoma
TIME: Tumor immune microenvironment
DDRPLS: Dedifferentiated retroperitoneal liposarcoma
TILs: Tumor-infiltrating lymphocytes
IHC: Immunohistochemistry
RNA-seq: RNA sequencing
DEGs: Differentially expressed genes
FFPE: Formalin-fixed and paraffin-embedded
TLS: Tertiary lymphoid structure
DCs: Dendritic cells
GO: Gene Ontology
KEGG: Kyoto Encyclopedia of Genes and Genomes
