# Supplementary material for: Immune characteristics of dedifferentiated retroperitoneal liposarcomas and the reliability of regional samples in evaluating their tumor immune microenvironments
Source: World J Surg Oncol. 2024 Jan 23;22:25. doi: 10.1186/s12957-023-03295-0 (PMC10804478; doi:10.1186/s12957-023-03295-0)
Supplement: Supplementary file 1 — Additional file 1: Supplementary Table S1. Antibody information. Supplementary Table S2. All immune markers corresponding genes. Supplementary Table S3. Correlation between immune markers and clinicopathological features of patients. [file 12957_2023_3295_MOESM1_ESM.docx]

**Supplementary Tables**

**Supplementary Table 1. Antibody information**

| Antibody name | Manufacturer | Article number | Dilution ratio |
| --- | --- | --- | --- |
| CD4 | abcam | ab133616 | 1:600 |
| CD8 | Affinity | AF5126 | 1:300 |
| Foxp3 | Affinity | AF6544 | 1:300 |
| CD20 | abcam | ab78237 | 1:300 |
| CD68 | Affinity | DF7518 | 1:300 |
| LAMP3 | Affinity | DF7099 | 1:300 |
| PD-1 | SAB | 24505 | 1:300 |
| PD-L1 | abcam | ab213524 | 1:300 |

**Supplementary Table 2. All immune markers corresponding genes**

| Markers | Corresponding genes | Reference (PMID) |
| --- | --- | --- |
| CD8^+^ T cell | CD8A, CD8B | 27765066 |
| CD4^+^ T cell | IGFBP4, ITM2A, AMIGO2, TRAT1, CD40LG, ICOS | 28104840 |
| Treg T cell | IL2RA, FOXP3, CTLA4, SLC35D1, GDPD3, CISH | 28104840 |
| B cell | BANK1, CD19, CD22, CD79A, CR2, FCRL2, IGKC, MS4A1, PAX5 | 27765066 |
| Macrophage | ADAP2, CSF1R, FPR3, KYNU, PLA2G7, RASSF4, TFEC | 27765066 |
| DC | CD1A, CD1B, CD1E, CLEC10A, CLIC2, WFDC21P | 27765066 |
| PD-1 | CD279,PDCD1 |  |
| PD-L1 | CD274 |  |
| TLS | CCL2, CCL3, CCL4, CCL5, CCL8, CCL18, CCL19, CCL21, CXCL9, CXCL10, CXCL11, CXCL13 | 21703392 |

**Supplementary Table 3. Correlation between immune markers and clinicopathological features of patients**

| Clinical features | CD4  (*P* value) | CD8  (*P* value) | Foxp3  (*P* value) | CD20  (*P* value) | CD68  (*P* value) | LAMP3  (*P* value) | PD-1  (*P* value) | PD-L1  (*P* value) | TLS  (*P* value) |
| --- | --- | --- | --- | --- | --- | --- | --- | --- | --- |
| Tumor size(cm) | 0.0001 | 0.0377 | 0.9672 | 0.1099 | 0.0098 | 0.2682 | 0.6401 | 0.0048 | 0.0009 |
| ≤20 |  |  |  |  |  |  |  |  |  |
| ＞20 |  |  |  |  |  |  |  |  |  |
| Age (y) | 0.6681 | 0.0193 | 0.0029 | 0.1070 | 0.0028 | 0.3513 | 0.3715 | 0.0439 | 0.7950 |
| ≤60 |  |  |  |  |  |  |  |  |  |
| ＞60 |  |  |  |  |  |  |  |  |  |
| Gender | 0.0003 | 0.0188 | 0.6888 | 0.0193 | 0.0300 | 0.1326 | 0.4720 | 0.0034 | 0.0044 |
| Male |  |  |  |  |  |  |  |  |  |
| Female |  |  |  |  |  |  |  |  |  |
| Diagnosis status | 0.1576 | 0.5894 | 0.0485 | 0.7053 | 0.6986 | 0.6817 | 0.5400 | 0.1959 | 0.0896 |
| Primary |  |  |  |  |  |  |  |  |  |
| Recurrent |  |  |  |  |  |  |  |  |  |
| Tumor number | 0.0001 | 0.0038 | 0.8586 | 0.0016 | 0.0007 | 0.0219 | 0.0624 | 0.0002 | 0.0001 |
| Single |  |  |  |  |  |  |  |  |  |
| Multiple |  |  |  |  |  |  |  |  |  |
