# Supplementary material for: Immune characteristics of dedifferentiated retroperitoneal liposarcomas and the reliability of regional samples in evaluating their tumor immune microenvironments
Source: World J Surg Oncol. 2024 Jan 23;22:25. doi: 10.1186/s12957-023-03295-0 (PMC10804478; doi:10.1186/s12957-023-03295-0)
Supplement: Supplementary file 2 — Additional file 2: Supplementary Figure 1. Immune cell constitution in DDRPLS using RNA-seq data. For most tumors, the distribution range of immune cells was narrow for different tumor regions. Each dot denotes the relative gene expression of a sample. DDRPLS, Dedifferentiated retroperitoneal liposarcoma; RNA-seq, RNA sequencing. Supplementary Figure 2. Immune cell constitution in the same region of DDRPLS. Very few immune cells were observed in DDRPLS. A: Immune cells in the same region of DDRPLS (IHC, represented by T3). B: Immune cell density in the same region of DDRPLS (represented by T3). Each dot denotes a tumor. C: Immune cell proportion in the same region of DDRPLS (represented by T3). Each dot denotes a tumor. DDRPLS, Dedifferentiated retroperitoneal liposarcoma; IHC, Immunohistochemistry. Supplementary Figure 3. Representative IHC images of immune cells in DDRPLS. Very few immune cells were observed in DDRPLS. IHC, Immunohistochemistry; DDRPLS, Dedifferentiated retroperitoneal liposarcoma. Supplementary Figure 4. Immune cell constitution in the same region of DDRPLS. Very few immune cells were observed in DDRPLS. A: Immune cell density in the same region of DDRPLS (represented by T3). B: Immune cell proportion in the same region of DDRPLS (represented by T3). DDRPLS, Dedifferentiated retroperitoneal liposarcoma. Supplementary Figure 5. Relationship between immune markers and tumor type in DDRPLS. Fewer immune markers were detected in multiple tumorsa single tumor. *p < 0.05, **p < 0.01, ***p < 0.001, ****p < 0.0001. DDRPLS, Dedifferentiated retroperitoneal liposarcoma; TLS, Tertiary lymphatic structure. Supplementary Figure 6. Relationship between immune markers and tumor size in DDRPLS. Immune markers were negatively correlated with tumor size in DDRPLS. DDRPLS, Dedifferentiated retroperitoneal liposarcoma; TLS, Tertiary lymphatic structure. Supplementary Figure 7. Correlation between IHC and RNA-seq data for the evaluation of TIME. RNA-seq was positivel [file 12957_2023_3295_MOESM2_ESM.docx]

**Supplementary Figures**


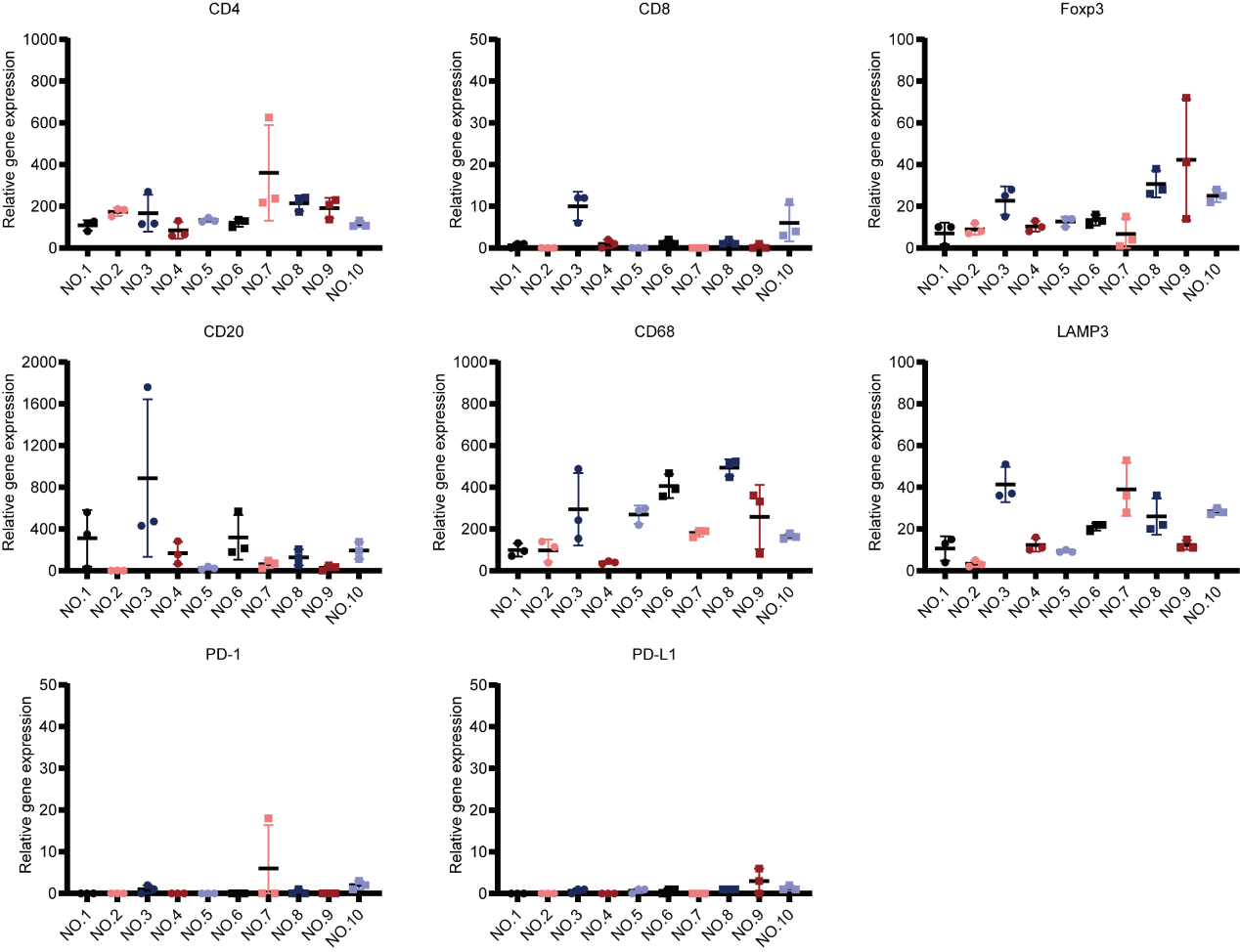


**Supplementary Figure 1. Immune cell constitution in DDRPLS using RNA-seq data**. For most tumors, the distribution range of immune cells was narrow for different tumor regions. Each dot denotes the relative gene expression of a sample. DDRPLS, Dedifferentiated retroperitoneal liposarcoma; RNA-seq, RNA sequencing.


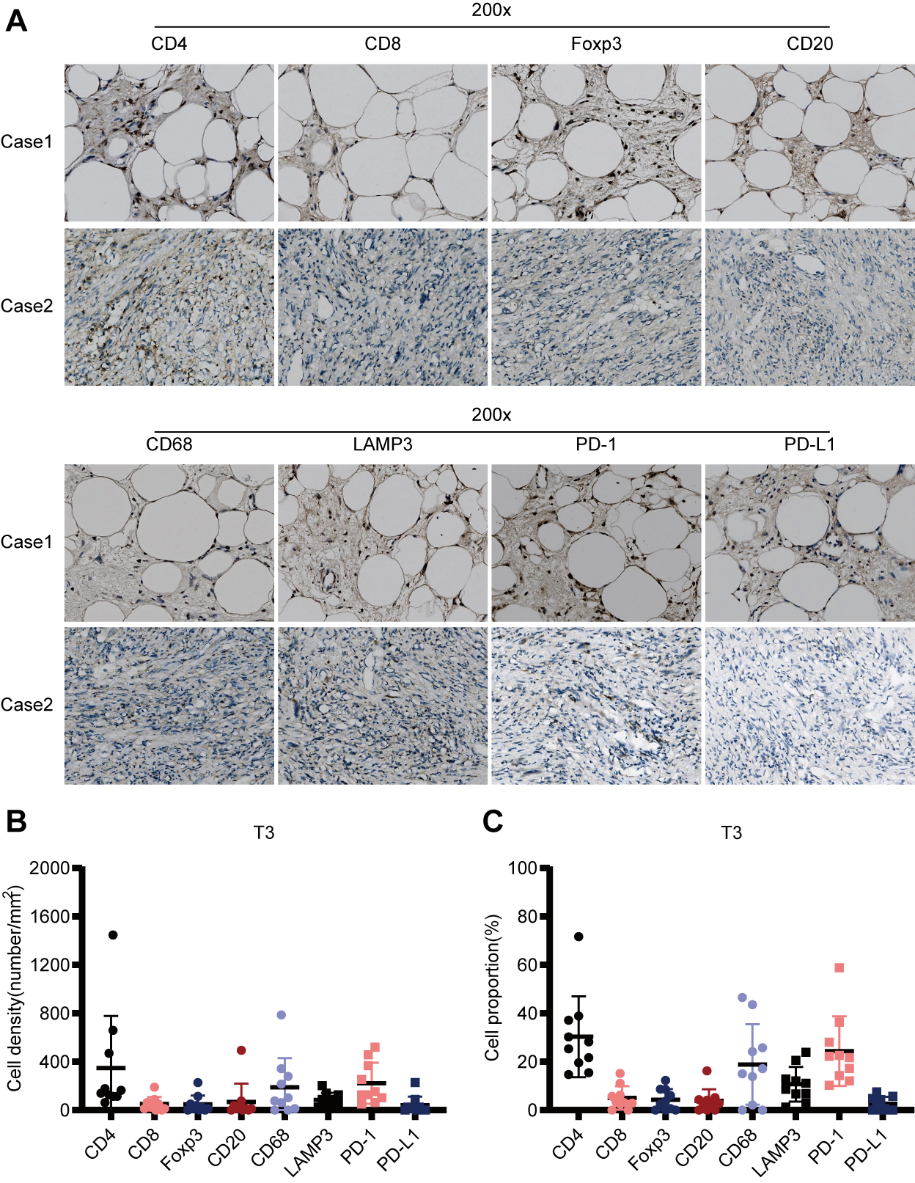


**Supplementary Figure 2. Immune cell constitution in the same region of DDRPLS.** Very few immune cells were observed in DDRPLS. **A:** Immune cells in the same region of DDRPLS (IHC, represented by T3). **B:** Immune cell density in the same region of DDRPLS (represented by T3). Each dot denotes a tumor. **C:** Immune cell proportion in the same region of DDRPLS (represented by T3). Each dot denotes a tumor. DDRPLS, Dedifferentiated retroperitoneal liposarcoma; IHC, Immunohistochemistry.


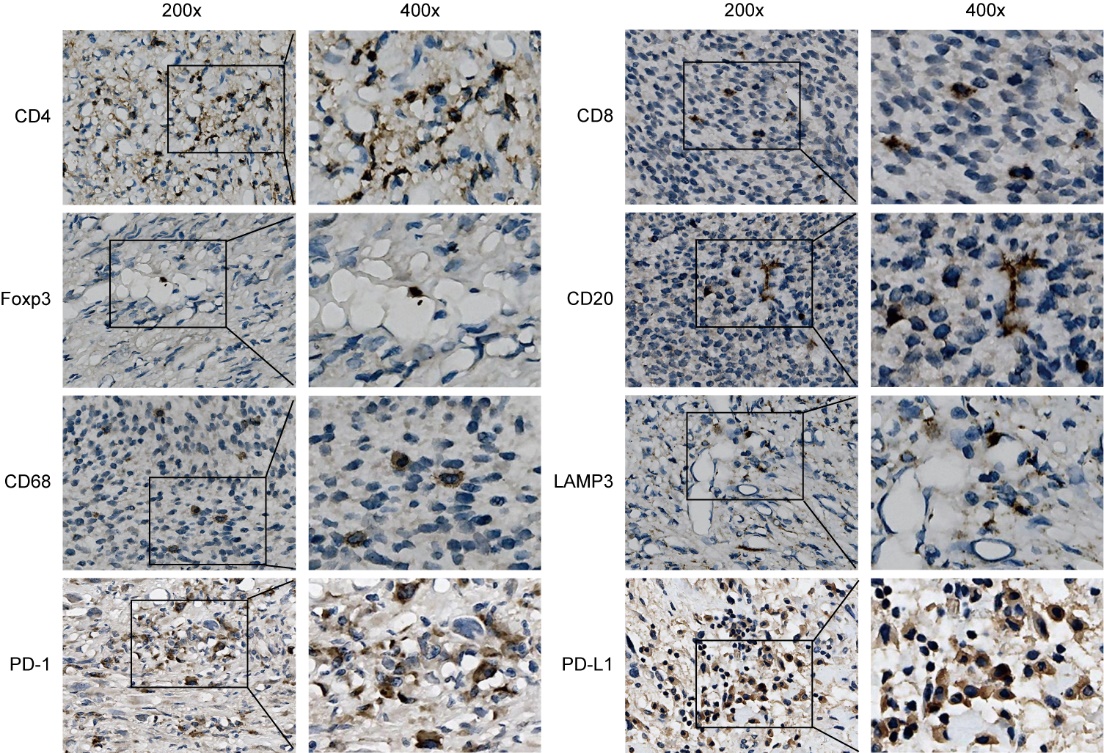


**Supplementary Figure 3. Representative IHC images of immune cells in DDRPLS.** Very few immune cells were observed in DDRPLS. IHC, Immunohistochemistry; DDRPLS, Dedifferentiated retroperitoneal liposarcoma.


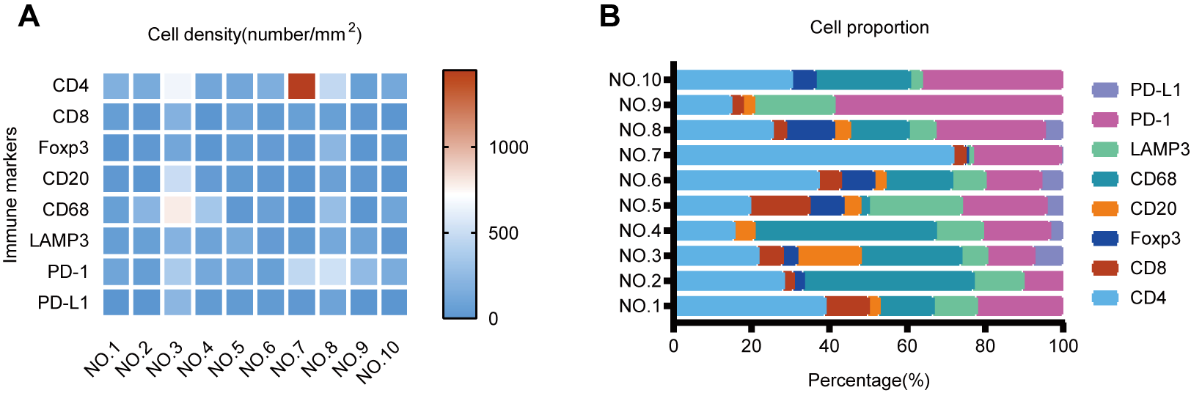


**Supplementary Figure 4. Immune cell constitution in the same region of DDRPLS.** Very few immune cells were observed in DDRPLS. **A:** Immune cell density in the same region of DDRPLS (represented by T3). **B:** Immune cell proportion in the same region of DDRPLS (represented by T3). DDRPLS, Dedifferentiated retroperitoneal liposarcoma.


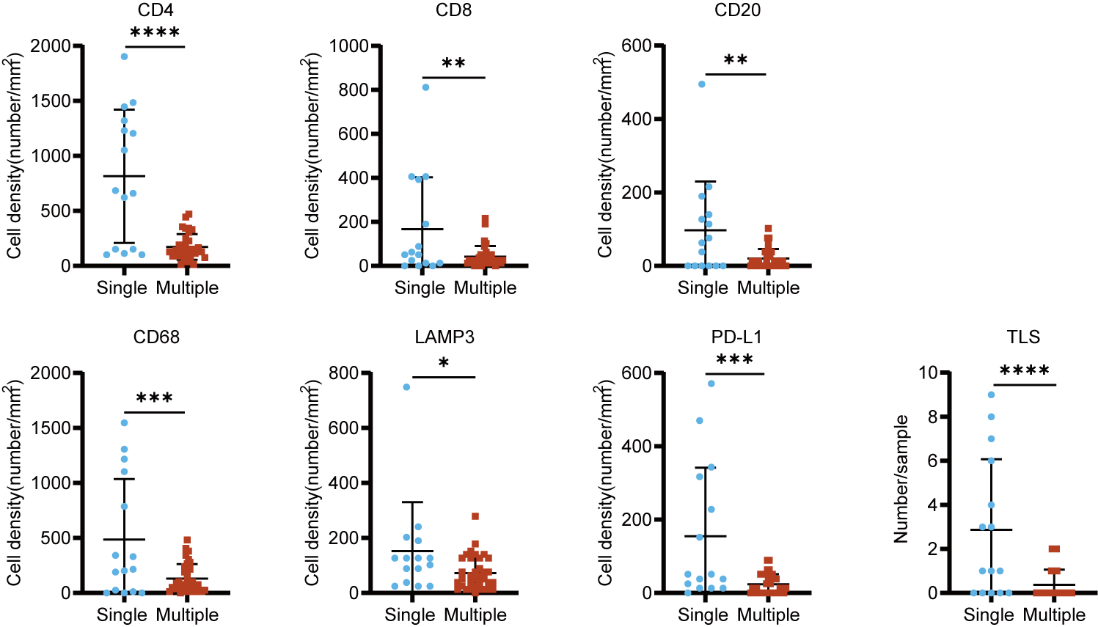


**Supplementary Figure 5. Relationship between immune markers and tumor type in DDRPLS.** Fewer immune markers were detected in multiple tumorsa single tumor. **p* < 0.05, ***p* < 0.01, ****p* < 0.001, *****p* < 0.0001. DDRPLS, Dedifferentiated retroperitoneal liposarcoma; TLS, Tertiary lymphatic structure.


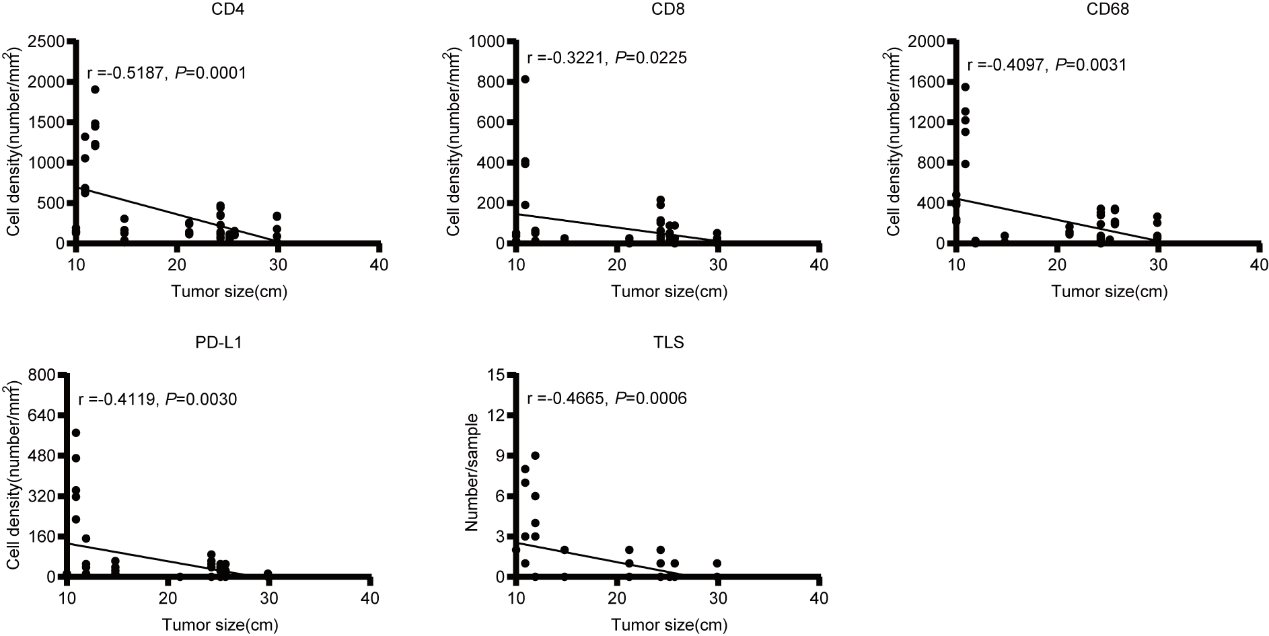


**Supplementary Figure 6. Relationship between immune markers and tumor size in DDRPLS.** Immune markers were negatively correlated with tumor size in DDRPLS. DDRPLS, Dedifferentiated retroperitoneal liposarcoma; TLS, Tertiary lymphatic structure.


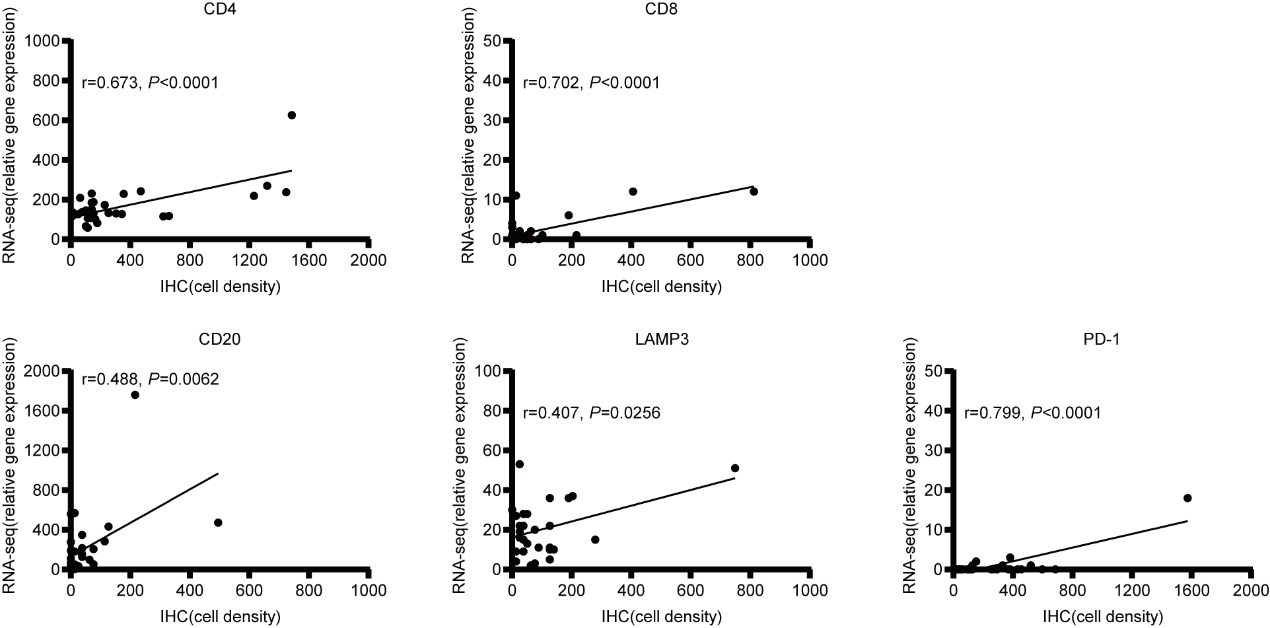


**Supplementary Figure 7. Correlation between IHC and RNA-seq data for the evaluation of TIME.** RNA-seq was positively correlated with IHC data for the evaluation of TIME. IHC, Immunohistochemistry; RNA-seq, RNA sequencing; and TIME, Tumor immune microenvironment.

**
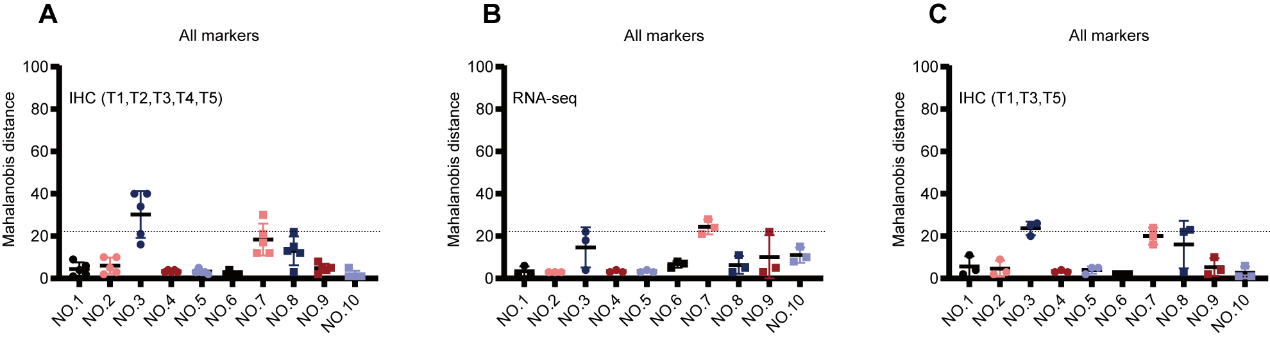
**

**Supplementary Figure 8. Consistency between IHC and RNA-seq for evaluating TIME.** IHC and RNA-seq data demonstrated consistent results for the evaluation of TIME. **A:** Mahalanobis distance of all regions (T1-5) used for IHC. **B:** Mahalanobis distance of all regions used for RNA-seq. **C:** Mahalanobis distance of regions (T1, T3, and T5) used for RNA-seq (detected by IHC). Each dot denotes the Mahalanobis distance of a region. Dotted lines denote the critical value of Mahalanobis distance. IHC, Immunohistochemistry; RNA-seq, RNA sequencing; and TIME, Tumor immune microenvironment.


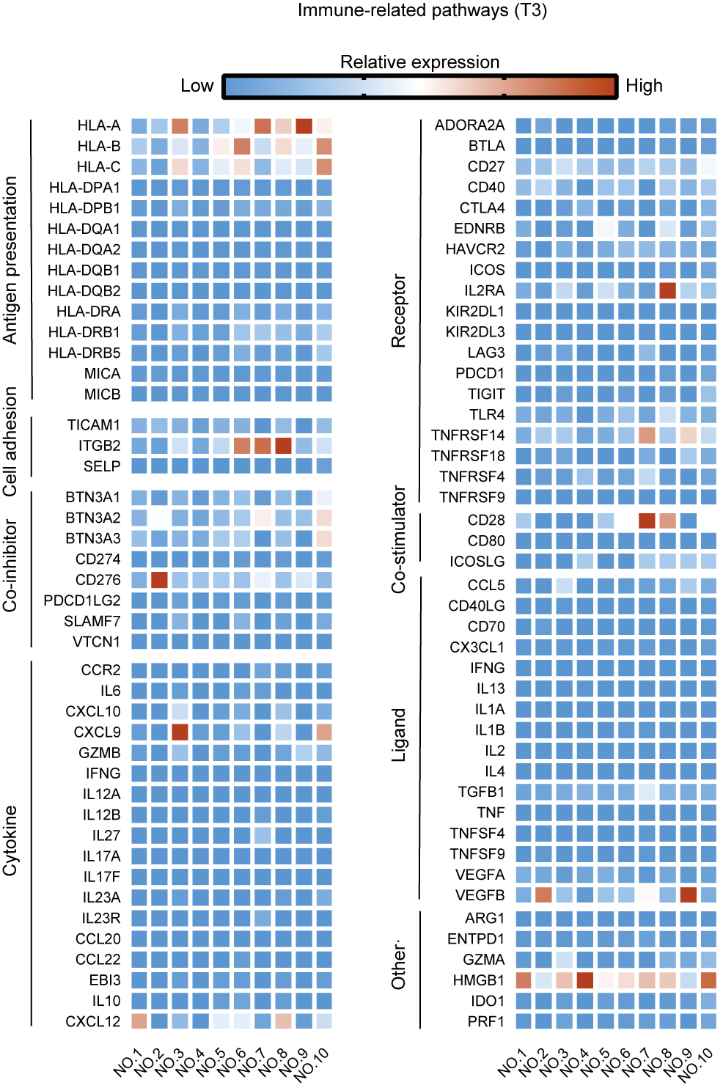


**Supplementary Figure 9. Relative expression of immune-related pathways in DDRPLS.** The relative expression of immune-related pathways in DDRPLS was low (represented by T3). DDRPLS, Dedifferentiated retroperitoneal liposarcoma.


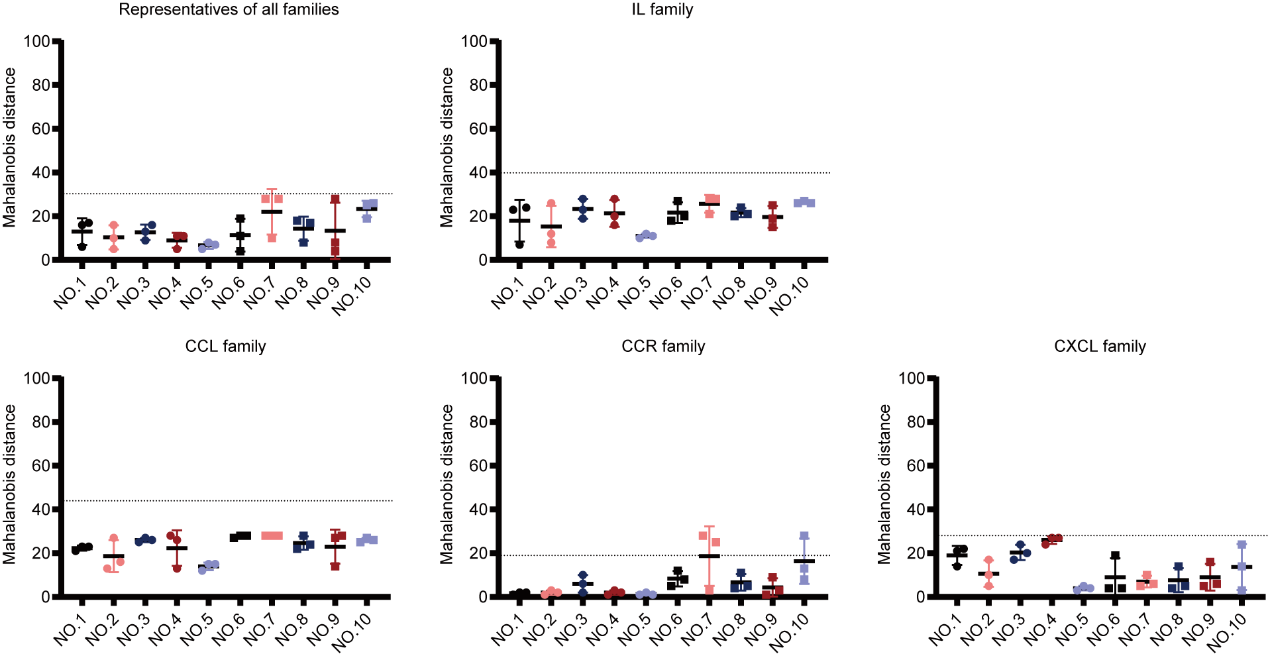


**Supplementary Figure 10. Similarity of cytokines in different tumor regions of using RNA-seq data alone.** Cytokines in different tumor regions were similar. Each dot denotes the Mahalanobis distance of a region. Dotted lines denote the critical value of Mahalanobis distance. RNA-seq, RNA sequencing.


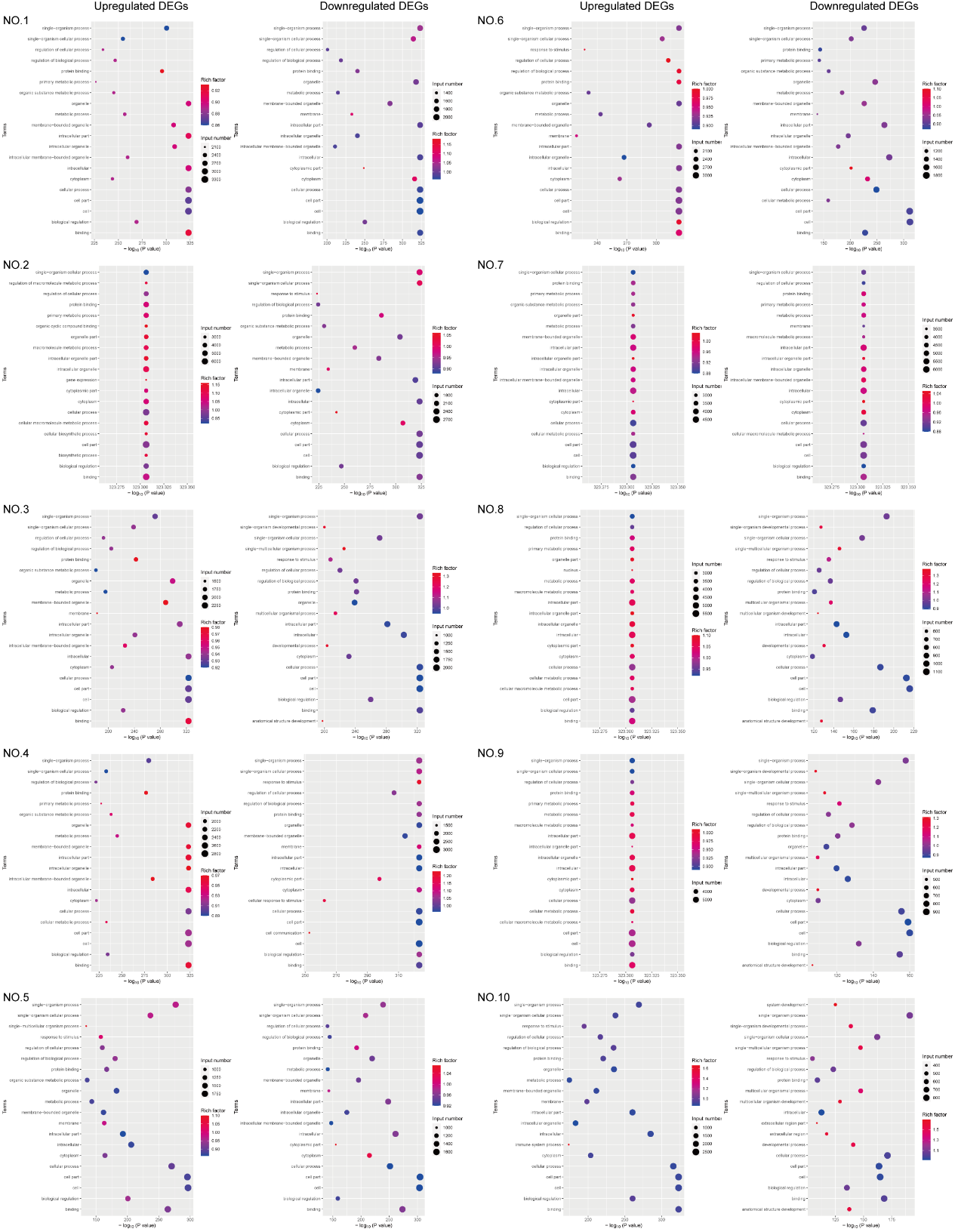


**Supplementary Figure 11. GO enrichment analysis of DEGs in DDRPLS.** Compared to paratumor tissues, DEGs in DDRPLS were significantly enriched in the intracellular part, intracellular, cellular process, cell part, cell, binding, and single organism process functions, with the most significant enrichment in the cell part and cell functions. GO, Gene Ontology; DEGs, Differentially expressed genes; DDRPLS, Dedifferentiated retroperitoneal liposarcoma.
